# CoExpNetViz: Comparative Co-Expression Networks Construction and Visualization Tool

**DOI:** 10.3389/fpls.2015.01194

**Published:** 2016-01-05

**Authors:** Oren Tzfadia, Tim Diels, Sam De Meyer, Klaas Vandepoele, Asaph Aharoni, Yves Van de Peer

**Affiliations:** ^1^Department of Plant Systems Biology, Vlaams Instituut voor BiotechnologieGhent, Belgium; ^2^Department of Plant Biotechnology and Bioinformatics, Ghent UniversityGhent, Belgium; ^3^Bioinformatics Institute Ghent, Ghent UniversityGhent, Belgium; ^4^Department of Plant Sciences and the Environment, Weizmann Institute of ScienceRehovot, Israel; ^5^Genomics Research Institute, University of PretoriaPretoria, South Africa

**Keywords:** co-expression, comparative genomics, networks, cytoscape, plants

## Abstract

**Motivation:** Comparative transcriptomics is a common approach in functional gene discovery efforts. It allows for finding conserved co-expression patterns between orthologous genes in closely related plant species, suggesting that these genes potentially share similar function and regulation. Several efficient co-expression-based tools have been commonly used in plant research but most of these pipelines are limited to data from model systems, which greatly limit their utility. Moreover, in addition, none of the existing pipelines allow plant researchers to make use of their own unpublished gene expression data for performing a comparative co-expression analysis and generate multi-species co-expression networks.

**Results:** We introduce CoExpNetViz, a computational tool that uses a set of query or “bait” genes as an input (chosen by the user) and a minimum of one pre-processed gene expression dataset. The CoExpNetViz algorithm proceeds in three main steps; (i) for every bait gene submitted, co-expression values are calculated using mutual information and Pearson correlation coefficients, (ii) non-bait (or target) genes are grouped based on cross-species orthology, and (iii) output files are generated and results can be visualized as network graphs in Cytoscape.

**Availability:** The CoExpNetViz tool is freely available both as a PHP web server (link: http://bioinformatics.psb.ugent.be/webtools/coexpr/) (implemented in C++) and as a Cytoscape plugin (implemented in Java). Both versions of the CoExpNetViz tool support LINUX and Windows platforms.

## 1. Introduction

A biological pathway is represented by a set of molecular entities (e.g., genes) that are involved in a given biological process and often interact with each other. Filling the gaps in our current knowledge with respect to biological pathways is a fundamental challenge. Although current insight into some biological pathways is substantial and useful for systems-level analyses, not all genes that participate in these pathways and affect their function are known and even in extensively studied model plants such as Arabidopsis and rice, many genes are still lacking experimental functional annotation (Rhee and Mutwil, 2014). Furthermore, many other biological pathways are still exhibiting major information gaps such as for instance those generating specialized metabolites (formerly known as secondary metabolites) in plants (Hansen et al., 2014; Tohge et al., 2014).

Advancements in computational approaches and robust statistical methods, along with the ever-increasing availability of transcriptomics data sets provide an excellent platform for gene discovery in unresolved or partly known pathways. Considering the premise that genes participating in the same biological process might posses a more similar expression pattern than expected by chance, co-expression is one of the most widely used functional gene discovery methods to fill gaps in metabolic pathways. Moreover, co-expression analysis allows the transfer of information from model (e.g., Arabidopsis, tomato, rice, maize, etc.) to non-model plant species (Stuart et al., 2003; Usadel et al., 2009; Heyndrickx and Vandepoele, 2012; Tzfadia et al., 2012; Movahedi et al., 2012; Itkin et al., 2013; Amar et al., 2014; Rhee and Mutwil, 2014).

So far, most reports on functional gene discovery via co-expression analysis in plants described the use of transcriptome data for a single species. Finding conserved co-expression patterns between orthologs across related plant species can provide a highly relevant list of candidate genes that potentially share similar functions and act in the same pathways (Hirai et al., 2005; Obayashi et al., 2007; Usadel et al., 2009; Mutwil et al., 2010, 2011; Movahedi et al., 2012; Hansen et al., 2014; Tohge et al., 2014). An example of using such a strategy was recently described for tomato and potato, where comparative co-expression information was utilized for constructing a co-expression network, leading to the discovery of a metabolic gene cluster related to the steroidal glycoalkaloids (SGAs) pathway (Itkin et al., 2013).

Several co-expression-based tools have been commonly used in plant research, such as ATED-II (Obayashi et al., 2007), PlaNet (Mutwil et al., 2011), GeneCAT (Mutwil et al., 2008), CORNET (De Bodt et al., 2012), Complex (Neotea et al., 2014), PODC (Ohyanagi et al., 2015) and Expressolog (Patel et al., 2015). However, most of these pipelines are limited to data from model systems, which limit their utility. In addition, none of these pipelines allow plant researchers to make use of their own (custom and/or unpublished) gene expression data for performing a comparative co-expression analysis and generate multi-species co-expression networks. Moreover, downstream analysis is usually hindered by the inaccessibility of the output files and networks.

**Figure 1:**
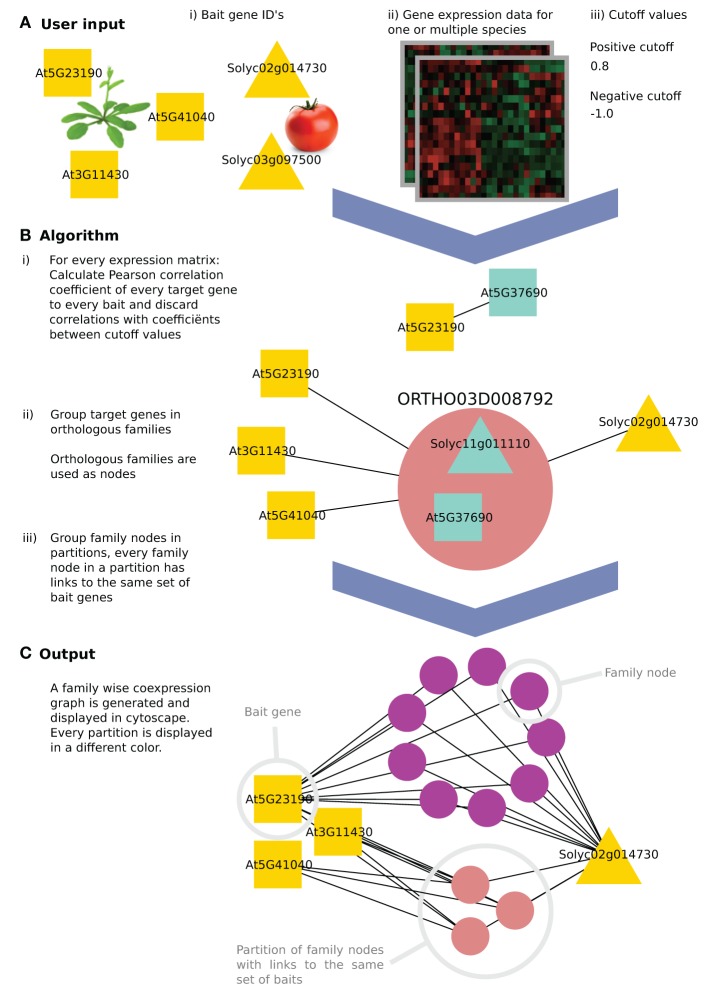
**The algorithm workflow of CoExpNetViz**. **(A)** The user input is (i) a number of genes of interest (the query or “bait” genes), along with (ii) gene expression data and (iii) two cutoff values. In this example, three *Arabidopsis thaliana* (squares) and two *Solanum lycopersicum* (triangles) bait genes are chosen. **(B)** CoExpNetViz will (1) calculate a correlation matrix for every species individually and translate correlations above the positive cutoff value or below the negative cutoff value into edges in a network. Then (2) all non-bait genes are grouped into gene families and (3) the families are ordered into the same partition if they share links with the same set of bait genes. **(C)** After running the analysis, the output can be visualized in Cytoscape.

Here, we introduce a user-friendly tool, called CoExpNetViz, that will allow biologists to use their own transcriptomics data generated in multiple species of their choice for cross species co-expression analysis. Co-ExpNetViz can use any number of queries or “bait” genes (from one or multiple species) that are known to be involved in the same biological process or pathway. CoExpNetViz can be used to search for new genes involved in a common process, or to find functional orthologs of the bait genes (Figure 1). The output includes files for visualizing the network in Cytoscape (Shannon et al., 2003), and correlation matrices for the given bait genes. Additionally, the user could apply network clustering (hubs) / GO enrichment/ network properties using other Cytoscape plug-ins.

Finally, to illustrate the utility of CoExpNetViz, we describe a case study in which we recreated the comparative co-expression network in tomato (*Solanum lycopersicum*) and potato (*Solanum tuberosum*) for finding SGAs related genes as performed earlier (manually) in Itkin et al. (2013).

**Figure 2:**
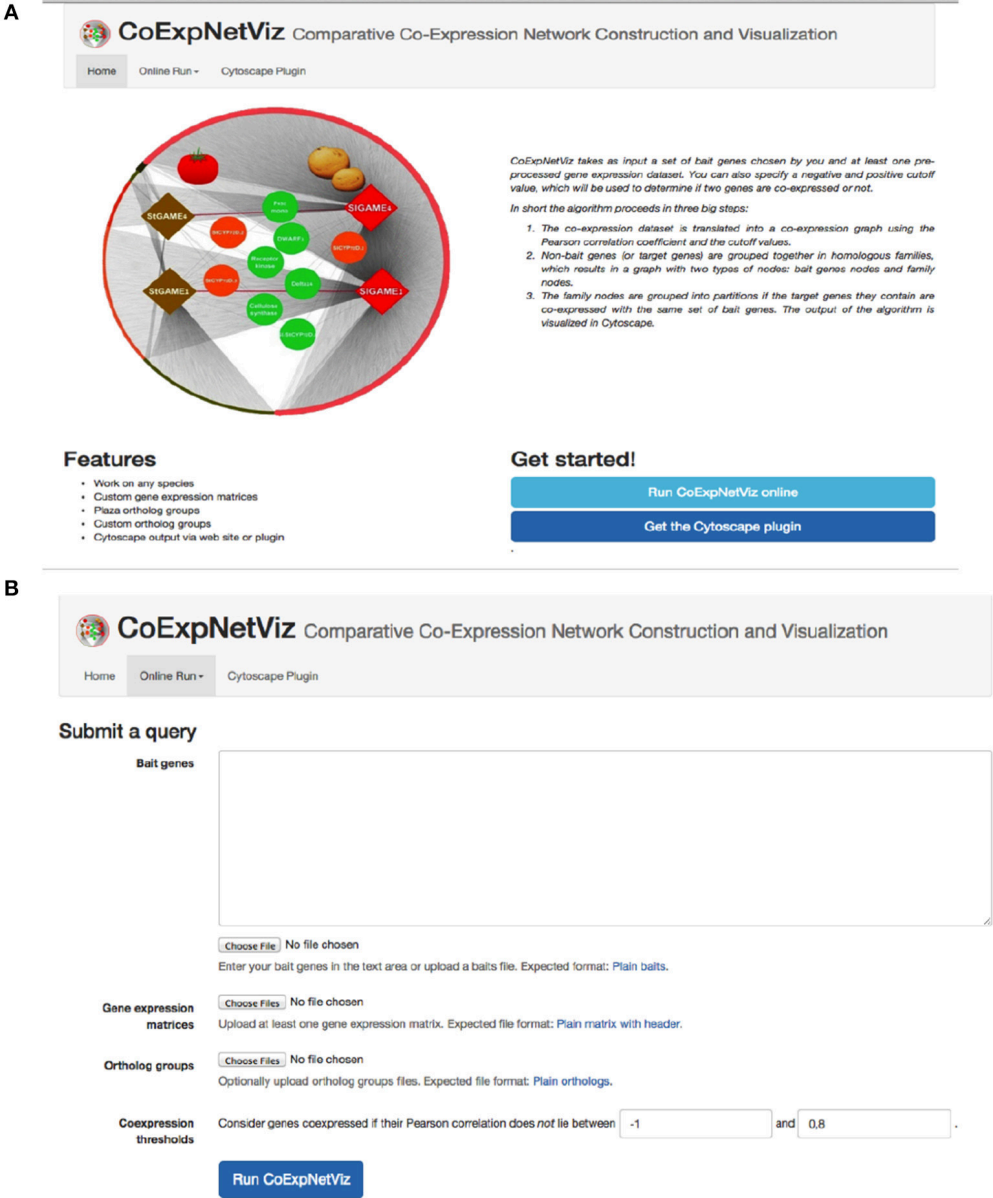
**Screen shots of the CoExpNetViz web interface**. **(A)** The home page of the CoExpNetViz containing links to: documentation for both developing the Cytoscap app Supplementary File 1, see Appendix A, and users (Supplementary File 1, see Appendix B), and for downloading the Cytoscape plugin. **(B)** The page for submitting a job to CoExpNetViz.

The graphical interface for the comparative co-expression construction console is available as both a PHP web server (http://bioinformatics.psb.ugent.be/webtools/coexpr/index.php; Figures 2A,B) and as a Cytoscape plug-in (see Appendix B; Figures B1–5, in Supplementary File 1).

## 2. Materials and methods

### 2.1. Algorithm

CoExpNetViz takes as input a set of “bait genes” from one or multiple species and one preprocessed expression data set per species. Normally, genes known to be involved in the same biological process are chosen as the baits. CoExpNetViz will then determine which gene families have co-expressed genes with these bait genes. The user specifies both negative and positive Pearson correlation coefficient cutoff values, which will be used to determine if two genes are co-expressed.

### 2.2. Correlation calculation

Many algorithms for calculating co-expression exist. Here we use Pearson correlation coefficient (PCC) which is the most popular algorithm used, and a custom Python implementation of mutual information (Song et al., 2012) as a measure for similarity between expression profiles. For a each species, a correlation value is calculated for every bait gene x = (x1, x2,…, xn) and every other gene y = (y1, y2, . . . , yn). By default we consider genes to be co-expressed if their correlation falls below the 5th or above the 95th percentile of a sample distribution of expression correlations based on the similarity between expression profiles for 4000 random genes (approximately 1,000^*^999^*^0.5 gene pairs). Bait genes that are not present in the species' data set are discarded. This step results in the generation of a correlation matrix of bait and target genes for each of the species analyzed (see steps B.1–3 in Figure 1).

### 2.3. Network construction

After computing the correlation matrices, the positive and negative cut-off values are (Vandepoele et al., 2009) used to translate *r*-values into edges in a graph where nodes represent the genes and edges represent a co-expression relationship.

An edge between two genes is retained only if the *r*-value is above the positive cutoff value or below the negative cutoff value (displaying negative correlation). Finally, genes that do not contain any edges are discarded (see step B.1 in Figure 1).

### 2.4. Grouping homologous genes

For grouping target (i.e., co-expressed) genes into homologous families, we used (sub)gene families as available in PLAZA (Proost et al., 2015). These gene families are the result of clustering genes based on sequence similarity using the Markov clustering based Tribe-MCL algorithm (Enright et al., 2014), followed by a post-processing algorithm to identify outliers. A gene is defined as an outlier if it shows sequence similarity to only a limited number of genes in the gene family. Subfamilies are then inferred from the Tribe-MCL families using the Ortho-MCL algorithm (Li et al., 2003). The PLAZA platform has separate databases for monocotyledonous and dicotyledonous organisms. Yet, there are 10 species that are present in both databases: some of these species were included to serve as a reference to link both databases while others function as out-groups. To allow CoExpNetViz to work with datasets from monocots and dicots simultaneously, the overlapping species in PLAZA have been used to create merged families that contain both monocotyledonous and dicotyledonous species.

All target genes that were retained in the previous step (network construction) are next grouped into one node if they belong to the same gene family. These new nodes, termed here “family nodes,” contain only genes that were present in the gene expression data and that are co-expressed with at least one bait gene. Using these family nodes, a new graph is created in which the nodes are either family nodes or bait genes and edges represent co-expression relationships. An edge is drawn between a family node and a bait gene if at least one gene in the family node is co-expressed with that bait gene (see step B.2 in Figure 1).

### 2.5. Grouping family nodes into partitions

A partition is a set of family nodes where every family node (but not necessary all genes in that family), is co-expressed with the same set of baits. Partitions are computed from the previously obtained graph by grouping them into sets, which share the same neighboring nodes (see step B.3 in Figure 1). For example, in Figure 1, the purple nodes are all co-expressed with At5g23190 and Solyc02g014730, while all the pink nodes are also co-expressed with At3g11430 and At5g41040 in addition to At5g23190 and Solyc02g014730.

### 2.6. The CoExpNetViz output

The CoExpNetViz web interface creates network files, which can be readily imported into Cytoscape. If the plug-in is available, the network will be loaded automatically into Cytoscape. Family nodes are displayed as circles with specific colors, where every circle represents one partition. Bait genes are displayed in white, and when using the web interface, are grouped in one circle at the top left. An advantage of the plugin compared to the web interface is that the plugin provides an enhanced layout algorithm to place the bait genes of different species into different circles. These circles are then placed at equal distances around the parti-tions containing the family nodes (Figure 1C).

### 2.7. Downstream analysis

Once the co-expression network is created and visualized in Cytoscape, users can take advantage of the plethora of plug-ins available in Cytoscape and that allows users to quickly and conveniently analyze different properties of the co-expression network. Here, we will mention only a few key features for a full list of plug-ins available in Cytoscape, we refer the reader to the Cytoscape user manual.

BiNGO (Maere et al., 2005) is a Cytoscape plugin to determine which Gene Ontology (GO) categories are statistically overrepresented in a set of genes or a subgraph of a biological network. BiNGO maps the predominant functional themes of a given gene set on the GO hierarchy, and outputs this mapping as a Cytoscape graph. Additionally, it supports a wide range of organisms. MCODE (Bader and Hogue, 2003) is another plugin, which finds clusters (highly interconnected regions) in a large network.

### 2.8. Implementation

The CoExpNetViz Cytoscape tool is written mainly in java (Perl/BioPerl and Python were also used for parsing files into the desired format; see Supplementary file 1 for detailed descriptions). The website was implemented in C++, Perl, MySQL and Apache, and supports all major browsers (tested on Linux and Windows systems). All source code and binaries are freely available to non-commercial users for download at http://bioinformatics.psb.ugent.be/webtools/coexpr/index.php. The CoExpNetViz Cytoscape plugin was written in Java/OpenJDK (http://openjdk.java.net). We used Maven Building for documenting and organization of the plugin (http://maven.apache.org), and OSGi for integrating the CoExpNetViz tool into the Cytoscape core program (http://www.osgi.org). For debugging and version control we used Git and GitHub (http://git-scm.com and https://github.com).

## 3. Results

**Figure 3:**
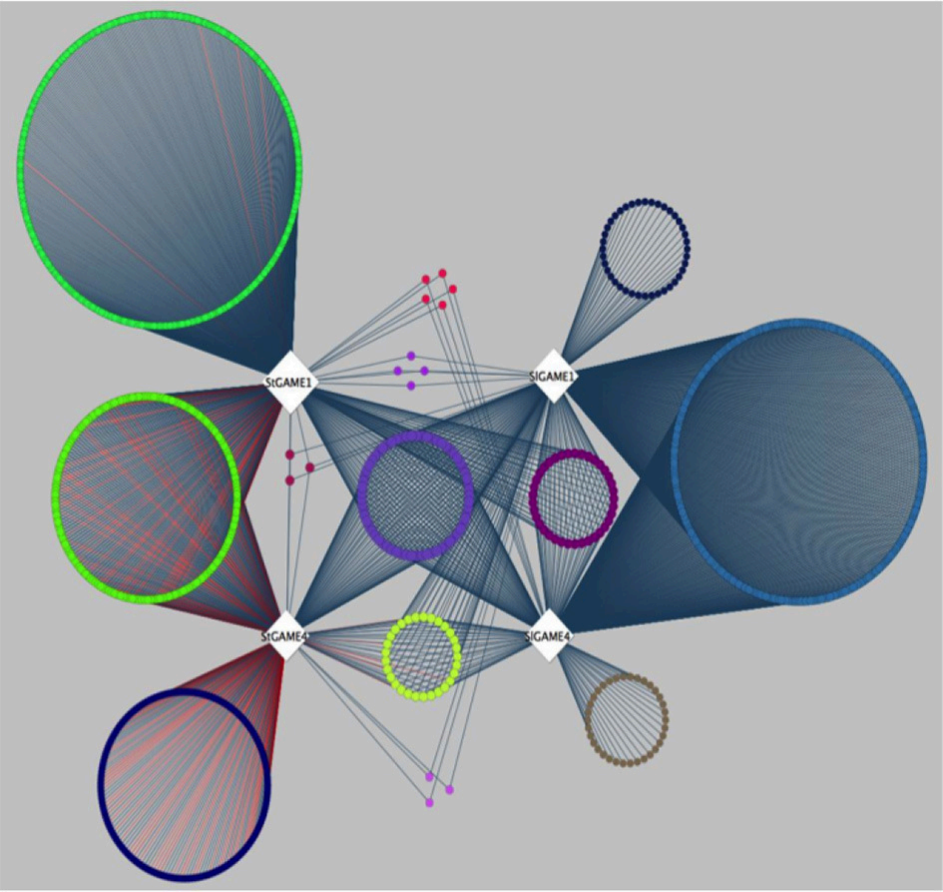
**Steroidal glycoalkaloids comparative gene co-expression network (from Itkin et al., 2013) reanalyzed by CoExpNetViz**. Edges connect co-expressed genes (nodes) exhibiting an *r*-value greater than 0.8 with the bait genes. The color of nodes of the co-expressed genes corresponds to the bait with which they were found to be co-expressed with. The light purple circle of nodes represent shared homologs of co-expressed genes for bait-genes from tomato (SlGAME1 and SlGAME4) and potato (StSGT1 and StGAME4). CoExpNetViz could successfully identify three glycosyltransferases (GAME10a, GAME17, and GAME18), a delta(24)-sterol reductase-like protein (GAME19), a BHLH transcription factor (GAME20) and a sterol reductase (GAME23).

A recent publication by Itkin et al. (2013), presented comparative co-expression analysis to discover new genes that participate in the SGAs biosynthesis pathway in species of the Solanaceae family. Itkin and colleagues conducted a co-expression analysis between two individual species, namely tomato (*Solanum lycopersicon*) and potato (*Solanum tuberosum*). Genes co-expressed with GAME1 and GAME 4 were determined in tomato. Orthologs of GAME1 and GAME4 were then determined in potato using BLAST. Next, co-expression analysis was carried out with GAME1 and GAME4 in tomato (SlGAME1 and SlGAME4) and using GAME1 and GAME4 in potato (StGAME1 and StGAME4). Careful examination of the co-expression network (see inner circle of Figure 3 in Itkin et al., 2013) combined with genomic clustering information and experimental validation led to the discovery of an operon-like cluster of genes involved in SGAs biosynthesis. To illustrate the features of CoExpNetViz for generating cross-species co-expression and its visualization, we used the bait genes SlGAME1, StGAME1, SlGAME4, and StGAME4, as in Itkin et al., 2013 as input for CoExpNetViz. By analyzing the co-expression network obtained (Figure 3), a number of known SGAs related genes could be retrieved. CoExpNetViz could successfully identify three glycosyltransferases (GAME10a, GAME17, and GAME18), a delta(24)-sterol reductase-like protein (GAME19), a BHLH transcription factor (GAME20) and a sterol reductase (GAME23). In addition, recent work showed that more genes that are co-expressed with the GAME genes in potato and tomato (yellow inner circle; see Figure 3) are involved in SGAs biosynthesis (Sawai et al., 2014). Interestingly CoExpNetViz provided additional candidate genes expressed with the GAME bait genes when compared to the candidate genes found in the co-expression network generated by Itkin et al., 2013. These additional candidate genes found by CoExpNetViz are likely to be a result of the utilization of the PLAZA gene families to determine orthologous genes which allow to account for many to many gene orthology mapping and therefore increase the candidate genes relevant to the pathway examined.

## 4. Discussion

The aim of CoExpNetViz is to identify genes that are co-expressed with as many of the query or bait genes as possible, preferably across multiple species. Being co-expressed with orthologous genes (across different species, rather than in just one species) makes the candidate genes more robust as they reflect an evolutionary conserved gene expression pattern. The approach used by CoExpNetViz to find such conserved co-expression relationships is to first find co-expressed genes within one species and then group these links across multiply species using the concept of homology. The CoExpNetViz could be further developed to offer more correlation methods. In addition we would like to make it possible to easily use species which are not in PLAZA to infer homology by parsing BLAST outputs.

## Author contributions

OT designed the project, analyzed the data and wrote the MS. TD developed the web tool, SD developed the Cytoscape plug-in, KV analyzed the data, wrote the MS. AA designed the project, analyzed the data, wrote the MS. YP analyzed the data, wrote the MS. All authors read, revised and approved the MS.

## Funding

The work in the AA lab was supported by the European Research Council grant SAMIT (no. 204575). We thank the Tom and Sondra Rykof Family Foundation for supporting the AA lab activity. AA is the incumbent of the Peter J. Cohn Professorial Chair. KV and YP acknowledge the Multidisciplinary Research Partnership “Bioinformatics: from nucleotides to networks” Project (no 01MR0310W) of Ghent University. YVdP also acknowledges support from the European Union Seventh Framework Programme (FP7/2007-2013) under European Research Council Advanced Grant Agreement 322739 “DOUBLE-UP.”

### Conflict of interest statement

The authors declare that the research was conducted in the absence of any commercial or financial relationships that could be construed as a potential conflict of interest. The Guest Associate Editor Aaron Fait declares that, despite having previously collaborated with the author Oren Tzfadia, the review process was handled objectively.

## Supplementary material

Supplementary File 1 **CoExpNetViz user and development manuals**.
