# Supplementary material for: CoExpNetViz: Comparative Co-Expression Networks Construction and Visualization Tool
Source: Front Plant Sci. 2016 Jan 5;6:1194. doi: 10.3389/fpls.2015.01194 (PMC4700130; doi:10.3389/fpls.2015.01194)
Supplement: Supplementary File 1 — CoExpNetViz user and development manuals. [file DataSheet1.PDF]

Supplementary file 1

Comparative Genomics Co-expression Networks

Authors:
Sam De Meyer & Dr. Oren Tzfadia

June 8, 2015

Contents

Contents i

List of Figures iii

List of Tables v

Glossary vii

1 Introduction 1
1.1 Function prediction via coexpression analysis 1
1.2 Overview of used computational techniques 3

A Development of CoExpNetViz 5
A.1 Compiling CoExpNetViz 6
A.1.1 Setting up a development environment 6
A.1.2 Compiling the source code 6
A.1.3 Running and testing CoExpNetViz 7
A.2 General outline 8
A.2.1 Basic structure of the application 8
A.2.2 The GUI classes 10
A.3 Guidelines concerning Cytoscape plugin development 10
A.3.1 Including other java packages 10
A.3.2 Adding third party libraries 11
A.3.3 Adding JUnit tests 12
A.3.4 Adding a layout algorithm 15
A.4 Development workflow 19
A.4.1 Debugging the plugin code 20
A.4.2 Stepping into the Cytoscape core program source code 21

B CoExpNetViz user manual 23
B.1 Installation 24
B.2 Usage of CoExpNetViz 24
B.3 The family wise coexpression graph 25
B.4 Web interface 25
B.5 File formats 25
B.6 How to submit bugs 29

Bibliography 31

List of Figures

A.1 Attaching the NetBeans debugger to CytoScape 21

B.1 CoExpNetViz input form 27
B.2 Overview of a family wise coexpression graph 27
B.3 Detail of the gene families 28
B.4 Detail of the bait genes 28
B.5 Linking gene families to the PLAZA website 28

List of Tables

1.1 Comparison of community accessible coexpression-based gene discovery tools 2

Glossary

API Application Programming Interface. vii, 17, 21, 22, 31

bait gene A gene of interest in the family wise coexpression graph for which you would like to discover conserved coexpression links. vii, viii, 3, 7, 9, 10

BAR the Botany Array Resource. 10

CSB.DB the Comprehensive Systems-Biology DataBase. 10

Cytoscape An open source software project for viewing and analyzing high throughput biological data. Cytoscape provides functionality for transforming high throughput biological data into networks (graphs). Network statistics and analysis of these graphs can be done from within Cytoscape. In addition, Cytoscape provides an Application Programming Interface (API) for writing plugins to extend the core program's functionality (Shannon *et al.*, 2003, Saito *et al.*, 2012). 3–5, 7, 9, 10, 17–22, 24, 25, 27–31

family node A node in the family wise coexpression graph that represents a set of genes which belong to the same gene family. Every gene in a family node must be coexpressed with at least one bait gene. vii, 7, 9

family wise coexpression graph A graph where nodes are either homologous gene families or bait genes. An edge between a bait gene and a family node represents a coexpression relationship between the bait gene and at least one other gene present in the family node. vii, 9

GEO Gene Expression Omnibus. 3

GO Gene Ontology. 2

Highest Reciprocal Rank A coexpression similartiy metric used by the PlaNet database (Mutwil *et al.*, 2011) based on the reciprocal rank of the Pearson Correlation Coefficient (PCC) between two genes. See `http://aranet.mpimp-golm.mpg.de/faq.html` for more information. vii, 4

HRR Highest Reciprocal Rank. 4, *Glossary:* Highest Reciprocal Rank

IDE Integrated Development Environment. 29, 30

JDK Java Development Kit. 18

OSGi Open Services Gateway Initiative. 19–21, 29

PCC Pearson Correlation Coefficient. vii, 4, 7, 10, 11

PLAZA A resource for plant comparative genomics. Trough a web interface, comparative genomic data of 37 plant species is provided, this includes Structural and functional annotations, classification into gene families, phylogenetic trees and information about genome organization among other things (Proost *et al.*, 2015). viii, 3, 5, 9, 12–14

PLAZA family A family of (putative) homologous protein encoding genes computed by PLAZA (Proost *et al.*, 2015). To compute gene families, the Tribe-MCL algorithm is used (Enright *et al.*, 2002) followed by a post processing algorithm to identify outliers. In short: an all vs all BLASTp (Altschul *et al.*, 1990) of all genes in the PLAZA database is performed to calculate sequence similarity scores for every possible gene pair. The sequence similarity scores are then normalized and used as a distance measure to perform Markov clustering (van Dongen, 2000), this is the Tribe-MCL algorithm as described by Enright *et al.* (2002). Finally the post processing algorithm is applied to remove outliers. The post-processing algorithm removes a gene from a gene family if it shows sequence similarity to only a limited number of genes in this family. 9

RBH Reciprocal best hit. 14

SCC Spearman's rank correlation coefficient. 10, 11

target gene Any gene that is present in the microarray data which was used in CoExpNetViz that is not a bait gene. 7, 9

TF transcription factor. 11

Chapter 1

Introduction

1.1 Function prediction via coexpression analysis

It is known that genes participating in the production of a certain metabolite tend to have more similar expression patterns than expected by chance. Thus, in order to discover how a plant produces a certain metabolite, one could use transcriptome analysis to gain information about the coexpression of enzymes and regulators correlated with the presence or absence of that metabolite (Usadel *et al.*, 2009a, Rhee and Mutwil, 2014). This way, genes of unknown function that are co-regulated with proteins that are known to be involved in the production of the metabolite of interest can be proposed as new candidates for experimental validation.

Comparative transcriptomics The practice of combining and integrating expression data from multiple species, known as comparative transcriptomics, adds another layer of information to transcriptome analysis, increasing the predictive power. There are a few problems associated with transcriptome data, one of them being that it is inherently noisy, increasing the number of both false positives and false negatives. Another problem is the failure to cover all genes, increasing the number of false negative results as well. The assumption used in comparative genomics is that, by looking at expression patterns that are conserved between orthologous genes, the noise is reduced while true regulatory interactions gain strength, as they are conserved across species. And indeed, several studies have shown that this is the case (Bergmann *et al.*, 2004, Stuart *et al.*, 2003).

Visualization and interpretation Comparative coexpression analysis often results in long lists genes that have varying degrees of similarity to each other in terms of expression patterns or sequence. A network (graph) can be used to visualize this data. In such a network, nodes represent genes while edges represent homology or coexpression relationships. One-to-one, one-to-many and many-to-many relationships become clear when using a network representation. Also, many types of information can be visualized on a network. For example edge color can be used to show the type of relationship while edge width can represent the degree of similarity between coexpressed genes and node color or shape can represent the species. Additionally, Gene Ontology (GO) annotations (Ashburner *et al.*, 2000), KEGG or Reactome pathway information (Kanehisa *et al.*, 2014, Croft *et al.*, 2014) or MapMan functional categories (Usadel *et al.*, 2009b) can be mapped onto the network to help the interpretation. Also, network statistics can be applied to ex-

**Tool:**	CoExpNetViz	PlaNet	GeneCat	CORNET
Comparative	Yes	Yes	Yes	Yes
Number of supported species	up to 5	7(1)	1 or 2	1 or 2
Own data	Yes	No(1)	No	Yes
User interface	Web interface and Cytoscape plugin	Web-interface(1)	Web-interface	Web-interface
Visualization	Cytoscape	Web-page and file import to Cytoscape is possible	Web-page	Cytoscape
Output files	Cytoscape network and attribute files	Cytoscape network and attribute files + .SVG images	None	Cytoscape network and attribute files
Similarity metric	PCC(2)	HRR(3)	PCC(2)	PCC(2)
Reference	Unpublished	Mutwil *et al.*, 2011	Mutwil *et al.*, 2008	De Bodt *et al.*, 2012

Table 1.1: Comparison of community accessible coexpression-based gene discovery tools for plants. (1) According to Mutwil *et al.* (2011) the PlaNet algorithm can be downloaded and tested locally with any microarray data. But currently (as of April 2015) the PlaNet downloads page (`http://aranet.mpimp-golm.mpg.de/download.html`) does not allow downloading the program. (2) Highest Reciprocal Rank, see `http://aranet.mpimp-golm.mpg.de/faq.html`. (3) Pearson Correlation Coefficient.

tract densely connected subgraphs which often contain genes belonging to same biological process.

Networks of individual genes as described above can become very large and complex. To resolve this, groups of homologous genes can be represented as one single node and edges can be filtered out if they are present in only one or a few species. This results in a 'family' network with only conserved coexpression links, where nodes represent families of homologous genes. Interpretation of such networks and interpretation of network topology is then less complicated.

CoExpNetViz CoExpNetViz is available as a Cytoscape plugin (Shannon *et al.*, 2003, Saito *et al.*, 2012) and as a web-tool. After choosing bait genes and microarray datasets in the Cytoscape plugin, the analysis is run and the resulting network is displayed immediately. Using the web tool, the user can download the Cytoscape files and import them manually into the program. Additionally the user could apply GO enrichment (using BiNGO for example (Maere *et al.*, 2005)) or analyze network properties utilizing other Cytoscape plugins.

By providing a user friendly Cytoscape plugin, it our goal to make gene function prediction trough comparative transcriptomics analysis accessible to plant researchers without specialized bioinformatics knowledge or programming skills.

1.2 Overview of used computational techniques

Programming techniques:

- Java/OpenJDK
  Writing the CoExpNetViz Cytoscape plugin
  (http://openjdk.java.net)
  - Git/GitHub
    Collaboration and version control
    (http://git-scm.com and https://github.com)
  - Maven
    Building, documenting and organization of the plugin
    (http://maven.apache.org)
  - OSGi
    Integrating the CoExpNetViz tool into the Cytoscape core program
    (http://www.osgi.org)
- Perl/BioPerl & Python
  Several small scripts to parse files into the desired format
  (https://www.perl.org, http://www.bioperl.org and https://www.python.org)
- R
  The first version of CoExpNetViz was written in R
  (http://cran.r-project.org/)
  - Bioconductor
    Downloading/manipulating gene expression datasets
    (http://www.bioconductor.org/)
  - ggplot2
    Creating graphs for this document
    (http://ggplot2.org/)

Tools used:

- Cytoscape
  Analyzing coexpression data
  (http://www.cytoscape.org)
- Genevesigator
  Exploring microarray data
  (https://genevestigator.com/gv)
- PLAZA
  Used for defining gene families in the CoExpNetViz Cytoscape plugin
  (http://bioinformatics.psb.ugent.be/plaza)

Appendix A

Development of CoExpNetViz

Foreword

This appendix contains guidelines to continue the development of the CoExpNetViz Cytoscape interface. There are two reasons why we included this appendix:

Firstly, as we strongly believe that open source code is great way to share and make scientific discoveries accessible to as many researchers around the world, we decided to include a detailed manual for developers. This manual will also help us in future extension efforts of developing the CoExpNetViz Cytoscape plugin.

Secondly, the Cytoscape wiki is far from perfect. Many code snippets on the wiki are incomplete, many are out of date (and thus plain wrong) and most of the Cytoscape Application Programming Interface (API) is not documented. This chapter contains some general guidelines and howto's that took our developers a lot time to find out. We have the intention to post some of these guidelines on the Cytoscape wiki to prevent other programmers from running into the same problems as we did.

A.1 Compiling CoExpNetViz

A.1.1 Setting up a development environment

CoExpNetViz, just as Cytoscape, is being developed at Github (see `https://github.com/`). The source code of CoExpNetViz can be found at `https://github.com/CoExpNetViz.git`, the Cytoscape source code is also available on Github, but is not required for building plugins (although it can be helpful, see **appendix A.4.2**).

Several programs have to be installed to develop a Cytoscape plugin:

First of all, the Java Development Kit (JDK) has to be installed. Cytoscape runs on java 1.6 and java 1.7. Java 1.8 might appear to work as well on first sight, but running Cytoscape on java 1.8 can cause many hard to track bugs that only appear after using it for a while. It is therefore recommended to use JDK 1.6 or JDK 1.7.

The second required program is Maven. This is a framework that helps in organizing and compiling a java project, and Cytoscape itself is also built using Maven. It can be downloaded from `http://maven.apache.org`. At least Maven 3.0 is required to successfully compile the code.

The third required program, as mentioned before, is Git. It is recommended to use the most recent version. Git can be downloaded from `http://git-scm.com`

The fourth and most obvious program is Cytoscape itself, although it should be noted that it is technically not required to have Cytoscape installed to write and compile a plugin. CoExpNetViz is written for version 3.1, therefore, to test the plugin, this version or higher should be installed. The program can be downloaded from `http://www.cytoscape.org`.

A.1.2 Compiling the source code

To get a local repository (copy of the source code) install Git and issue the following command in the terminal:

```
$ git clone https://github.com/SamDM/CoExpNetViz.git
```

This will download the source code into a child directory of the directory from where the command was used. The newly created directory is called CoExpNetViz, and contains one child directory, also called CoExpNetViz, along with one hidden directory: the git directory. It also contains a README file and a hidden file: `.gitignore` (there are many Git tutorials online that explain what these files and directories are for).

After downloading the source code for the plugin, move two directories down in the newly created folder, there you will find the `pom.xml` file, this a file that tells Maven how the plugin should be compiled and what dependencies are required to compile it. In order to compile the plugin, move to the directory containing the `pom.xml` file and run `mvn clean install` as shown below (make sure you have working internet connection before trying this):

```
$ cd CoExpNetViz/CoExpNetViz
$ mvn clean install
```

Maven will now automatically download all the dependencies that are required to compile the plugin, then it will automatically compile the plugin, after which it will automatically run JUnit tests. When running this command for the first time, it might take up to a minute to compile the program. Compiling it again later should not take more than ten seconds. If the plugin was compiled successfully, something that looks like the following can be seen in the terminal:

```
[INFO] ------------------------------------------------------------------------
[INFO] BUILD SUCCESS
[INFO] ------------------------------------------------------------------------
[INFO] Total time: 9.677 s
[INFO] Finished at: 2015-04-19T16:24:01+02:00
[INFO] Final Memory: 32M/221M
[INFO] ------------------------------------------------------------------------
```

A.1.3 Running and testing CoExpNetViz

If the build was successful, the compiled plugin, called `CoExpNetViz-1.0-SNAPSHOT.jar` can be found in the `target` folder. To test the plugin, copy it to your Cytoscape apps folder. This folder is usually located in a subdirectory of the `CytoscapeConfiguration` folder, which can be found in the location were Cytoscape was installed. The full path is:

```
# replace $CYTOSCAPE_HOME by the cytoscape intallation folder
$CYTOSCAPE_HOME/CytoscapeConfiguration/3/apps/installed/
```

Once the `.jar` file is copied to the mentioned folder, launch Cytoscape, if everything went well, the *Apps* menu should now contain an entry called CoExpNetViz, click the entry to launch the plugin.

To avoid having to copy the `.jar` every time you make a change to the source code, you can (when using a Unix system) symlink the `.jar` in the `target` folder to the Cytoscape `apps/installed` folder. Whenever the plugin is recompiled, Cytoscape will detect that the symlink in the apps folder is updated, and it will reload the newly compiled plugin automatically.

It is recommended to run Cytoscape from the command line, this gives you access to the command line interface of Cytoscape, where you can manually install and load plugins as well as other parts of Cytoscape. The console gives access to many other aspects of Cytoscape, in addition java exceptions are printed to the console. Cytoscape can be run from the command line as follows:

```
# change 'x' to the appropriate version
# make sure cytoscape.sh is executeable
$ ./Cytoscape_v3.x.x/cytoscape.sh
```

It is important to know that Cytoscape is a collection of smaller parts called “bundles”, which can be loaded and replaced while the programming is running. This is possible trough the Open Services Gateway Initiative (OSGi) framework, Cytoscape is essentially nothing more than a collection of OSGi bundles, and every plugin by itself is also a OSGi bundle. Incorrect configurations for the apache felix plugin in the `pom.xml`

(**appendix A.3.1**) can result in a failure to activate the compiled plugin in Cytoscape. If this is the case, run the command `list` in the Cytoscape console, this will show all bundles, their ID and their state. Then run the command `start x` (replace x with the ID of the plugin) to manually start the plugin, this will print an error message giving information about why the plugin was not activated. More detailed information is also written to a log file located at:

```
# replace $CYTOSCAPE_HOME by the cytoscape intallation folder
$CYTOSCAPE_HOME/CytoscapeConfiguration/3/framework-cytoscape.log
```

Therefore, if the plugin cannot be activated, looking at this file can show the cause of the problem. For some good advice on an efficient development workflow, see also **appendix A.4**.

A.2 General outline

CoExpNetViz is being developed in two parts, one part is the Cytoscape interface, which is discussed in this document, another part is the web server. The Cytoscape interface provides the user with a form where gene expression datasets can be chosen and parameters for the coexpression analysis can be specified. The settings and files are then sent to the server, which will run the actual algorithm. The response is sent back to the Cytoscape plugin, which will read the network files and node attribute files, convert them into a network and apply the layout algorithm and visual style.

There are two reasons why the application is split into a local Cytoscape plugin and a web server. The first reason is that the idea of making a Cytoscape plugin arose after development of the core algorithm had been started in C++. Cytoscape is programmed in java, which is a cross platform language, and the Cytoscape app installer does not support platform specific downloads. This means, in order to make a plugin that can run locally, compiled versions of the C++ core for all platforms should be embedded in the plugin jar, which would make the jar extremely big. Another solution would be to let the user manually download the platform specific C++ core and somehow integrate it in the plugin, which is not user-friendly. Maybe the best solution would be to reprogram the C++ core in java. We opted for the second best, solution: to run the C++ core on a web server. An advantage of this approach is that a user can also make use of the web server by itself to run the analysis, without the requirement of any installed programs, lowering the barrier to try out the tool. Another advantage is that by using a web server, big gene family files do not have to be included in the plugin jar, lowering the download size. The standalone web application is available at `http://bioinformatics.psb.ugent.be/webtools/coexpr`.

A.2.1 Basic structure of the application

The Cytoscape plugin code is divided into a number of packages. These packages together with embedded dependencies form an OSGi bundle which can be loaded into Cytoscape. The starting point of the application is `CyActivator.java` (which extends `AbstractCyActivator`) in the package `be.samey.internal`. When Cytoscape is loaded, the `start` method is invoked, any services the plugin provides are specified in this method. There are three services provided by CoExpNetViz: the app GUI, which is located in the

Apps menu, a layout algorithm and an event listener to run code on certain events that happen in the core program. The menu action, which launches the CoExpNetViz GUI, is invoked by the `actionPerfomed` method (inherited from `AbstractCyAction`) of the `MenuAction` class.

To summarize, there are two important entry points into the code, firstly, there is the `CyActivator` class, whose `start` method is run when Cytoscape is started. Secondly, there is the `MenuAction` class, whose `actionPerfomed` method is run when the user clicks the CoExpNetViz entry in the *Apps* menu.

The CyAppManager, CyModel and CyServices There are three other classes of interest in the `be.samey.internal` package:

- One instance of `CyAppManager` is created in the `CyActivator` `start` method and is central to the plugin. This class acts a as central control point: it provides methods to get the settings directory, run the coexpression analysis on the web server and to get IO helper classes. In addition, the `CyAppManager` has references to the `CyModel` and the `CyServices`. The same `CyAppManager` instance is passed on to many parts of the plugin.
- The `CyModel` is passed as an argument to the constructor of the `CyAppManager`. The `CyModel` has fields with getters and setters, which are used to keep track of the application state.
- The `CyServices` class is also passed as an argument to the `CyAppManager` constructor. This class has fields containing the Cytoscape model classes, along with getters and setters for those fields. Instances of the Cytoscape model classes can only be obtained by the `CyActivator`, by wrapping all these model classes in a `CyServices` object, they are more easily passed around to other parts of the app.

When an object has a reference to the `CyAppManager`, it can get information about the application state trough the `CyModel`, and it can invoke Cytoscape actions trough the `CyServices`.

Running the coexpression analysis The `runAnalysis` method in the `CyAppManager` is responsible for collecting the data specified by the user, sending it to server, getting back the response and finally displaying the network with the correct layout and visual style. Uploading files to the server happens trough a `http POST`, which expects `multipart/mixed` form data. The fields of the form data are:

- `baits`: plain text containing the baits separated by whitespace
- `matrix0` / `matrix1` / `matrix2` / `matrix3` and `matrix4`: the gene expression files
- `positive_correlation`: a decimal number in plain text specifying the positive cutoff value
- `negative_correlation`: a decimal number in plain text specifying the negative cutoff value
- `orthologs0` / `orthologs1` / `orthologs2` / `orthologs3` and `orthologs4`: the gene family files

The connection with the server is handled by the `ServerConn` class in the package `be.samey.io`. The multipart entity is sent to http://bioinformatics.psb.ugent.be/webtools/coexpr/index.php with two additional url parameters: `__controller=api` and `__action=execute_job`. This allows the server side program to differentiate between the plugin requesting an analysis and a user accessing the web tool.

A.2.2 The GUI classes

When the CoExpNetViz entry in the *Apps* menu is clicked, an instance of `GuiManager` (in the package `be.samey.gui`) is created. This class is the central control point for the GUI, and has references to model classes which keep track of the GUI state. When the `GuiManager` is initialized, the settings are read and the GUI is created and shown.

There are three packages for the gui: Firstly, there is `be.samey.gui`, which has the `GuiManager` and two classes that build *swing* components that together form the GUI. Secondly the package `be.samey.gui.model` has a two model classes that keep track of the GUI state. Thirdly, the `be.samey.gui.controller` package has classes that control button actions.

Adding a new GUI element To add a new element to the GUI, for example to the `InpPnl`, add the swing components (a `JButton`, `JLabel`, etc.) in the `InpPnl` constructor. Then create a controller class in the `be.samey.gui.controller` package which has the code to execute when an action on the new GUI element is performed. The controller class should extend `AbstrController.java`. Finally, set the controller class as a listener to the new GUI element in the `initGui` method of the `GuiManager`.

A.3 Guidelines concerning Cytoscape plugin development

Many of the following sections are about setting up the `pom.xml` file, and more specifically, setting the OSGi options. For a detailed explanation, see `http://felix.apache.org/documentation/subprojects/apache-felix-maven-bundle-plugin-bnd.html`

A.3.1 Including other java packages

When a new plugin is created from scratch using the `org.cytoscape.archetypes:cyaction-app` Maven archetype (see *Creating_an_OSGi_Bundle_Cytoscape_3_App* in the Cytoscape wiki), a basic `pom.xml` file is created. In the `plugins` tag of this file, the OSGi setup is specified by the `apache.felix` plugin. The default configuration looks like this:

```
...
<plugin>
  <groupId>org.apache.felix</groupId>
  <artifactId>maven-bundle-plugin</artifactId>
  <version>2.3.7</version>
  <extensions>true</extensions>
  <configuration>
    <instructions>
      <Bundle-SymbolicName>${bundle.symbolicName}</Bundle-SymbolicName>
```

```
      <Bundle-Version>${project.version}</Bundle-Version>
      <Export-Package>${bundle.namespace}</Export-Package>
      <Private-Package>${bundle.namespace}.internal.*</Private-Package>
      <Bundle-Activator>${bundle.namespace}.internal.CyActivator</Bundle-Activator>
    </instructions>
  </configuration>
</plugin>
...
```

Two tags in this plugin are especially important: the `Export-Package` and the `Private-Package` tags. The first tag specifies which packages should be exported by the bundle, any packages that are not exported are unavailable at runtime. To export all the packages of the plugin during runtime, change the line to:

```
<Export-Package>${bundle.namespace}.*</Export-Package>
```

The second tag (`Private-Package`) specifies which packages should not be exported during runtime, but should still be included in the bundle. The `Export-Package` tag takes precedence over `Private-Package` tag.

A.3.2 Adding third party libraries

To add a dependency to the plugin, add to following lines to the `pom.xml` in the `instructions` tag of the felix plugin configuration:

```
<Embed-Dependency>*;scope=!provided</Embed-Dependency>
<Embed-Transitive>true</Embed-Transitive>
<Import-Package>*;resolution:=optional</Import-Package>
```

The `Embed-Dependency` tag specifies which jars should be embedded in the plugin. By using the '*' character, all jars in the `dependencies` tag of the `pom.xml` are included, this means the Cytoscape APIs will also be included which is not necessary. To prevent this, `;scope=!provided` is added, now all jars will be included except for the ones which have the `provided` scope. All Cytoscape API jars are provided by the Cytoscape program, therefore, all Cytoscape API jars must be marked as `provided` in the `pom.xml` by adding `<scope>provided</scope>` as follows:

```
<dependency>
  <groupId>org.cytoscape</groupId>
  <artifactId>service-api</artifactId>
  <version>3.1.0</version>
  <scope>provided</scope>
</dependency>
```

The `Embed-Transitive` tag tells OSGi to embed transitive dependencies (dependencies of your dependencies) as well. Finally the `Import-Package` tag tells OSGi to embed all packages specified in the `pom.xml` (because of '*'), but the `resolution:=optional` will prevent embedding a package which has `optional` set to `true` if it is never imported by any other package. This way, all the packages of dependencies which have `optional` set to `true` will

not be embedded in the plugin `.jar` file if they are not needed. To make a dependency optional, add the line `<optional>true</optional>` as in the example below:

```
<dependency>
  <groupId>org.apache.httpcomponents</groupId>
  <artifactId>httpclient</artifactId>
  <version>4.4</version>
  <optional>true</optional>
</dependency>
```

Conclusion To add a third party library, 1: add the three lines mentioned at the beginning of this section, 2: set the scope of all Cytoscape API dependencies to `provided` and 3: make all third party dependencies `optional`. After doing this, the packages can be imported from within the plugin code and their classes will be available for use from within the plugin code.

Notes

- The approach explained here will only work for libraries that are available in the Cytoscape Maven repositories or the central Maven repositories (`http://mvnrepository.com`).
- Even if a certain third party library is already embedded in the Cytoscape core program (as is the case for `httpcomponents`), it is still recommended to embed it again in a plugin. Otherwise, a new Cytoscape release, which uses different versions of these third party libraries, can cause the plugin to break.

A.3.3 Adding JUnit tests

To use Junit tests, two extra dependencies, `junit` and `mockito` are required (see also **appendix A.3.2**). As these dependencies are only required during testing, they should be given the `test` scope. This can be done as follows:

```
<dependency>
  <groupId>junit</groupId>
  <artifactId>junit</artifactId>
  <version>4.10</version>
  <scope>test</scope>
</dependency>
<dependency>
  <groupId>org.mockito</groupId>
  <artifactId>mockito-all</artifactId>
  <version>1.10.19</version>
  <scope>test</scope>
</dependency>
```

Also, the following line should be changed in the felix setup in the `pom.xml`:

```
<Embed-Dependency>*;scope=!provided</Embed-Dependency>
```

to:

```
<Embed-Dependency>*;scope=!provided|test</Embed-Dependency>
```

As this will prevent the test dependencies to be embedded in the plugin `.jar` file.

Writing a JUnit test It is recommended to follow the default Maven folder structure when creating JUnit tests. For example if the class to test is found at:

```
$PLUGIN_ROOT/src/main/java/com/something/package_name/ClassName.java
```

then the test class should be found at:

```
$PLUGIN_ROOT/src/test/java/com/something/package_name/ClassNameTest.java
```

Also, any resources used by test classes should be found in this folder, or a subdirectory of this folder:

```
$PLUGIN_ROOT/src/test/resources/
```

The test class is then located in the `package_name` package in the `test` folder, it should have the following minimal layout:

```
package com.something.package_name;

import org.junit.After;
import org.junit.AfterClass;
import org.junit.Before;
import org.junit.BeforeClass;
import org.junit.Ignore;
import org.junit.Test;
import static org.junit.Assert.*;
// add other imports if needed

public class ClassNameTest {

    public ClassNameTest() {
    }

    // implement methods setUpClass(), tearDownClass(), setUp()
    // and tearDown() if necessary

    /**
     * Test of firstMethod method, of class className
     */
    @Test
    public void testFirstMethod() {
        // add assertions here
    }

    // tests of other methods here
}
```

Accessing resources is usually done with the `getResource()` method of some class loader. When using the default maven layout (as described above), any test resources will automatically be added to the search path of the `getResource()` method. An example of getting a test resource:

```
/**
 * Some test method in a test class
 */
public void testSomeMethod() {
  // only works if the default Maven file structure is used, e.g. the
  // the resource used here is located at:
  // $PLUGIN_ROOT/src/test/resources/someResource.foo
  URL url = getClass().getClassLoader().getResource("someResource.foo");
  InputStream is = url.openStream();
  // now the contents of the resource can be accesed with "is"
}
```

Using Cytoscape objects in JUnit tests Add the following to the `pom.xml` to use Cytoscape objects in JUnit tests:

```
<dependency>
  <groupId>org.cytoscape</groupId>
  <artifactId>model-impl</artifactId>
  <version>3.1.0</version>
  <type>test-jar</type>
  <scope>test</scope>
</dependency>
<dependency>
  <groupId>org.cytoscape</groupId>
  <artifactId>model-impl</artifactId>
  <version>3.1.0</version>
  <scope>test</scope>
</dependency>
```

Then, to get an instance of a `CyNetwork` object for example:

```
package com.something.package_name;

import org.cytoscape.model.CyNetwork;
import org.cytoscape.model.NetworkTestSupport;
// + JUnit imports and other packages if needed

public class ClassNameTest {

    // Constructor, setUp(), tearDown(), etc.

    /**
     * Some test method in a test class
     */
    public void testSomeMethod() {
        NetworkTestSupport nts = new NetworkTestSupport();
        CyNetwork cn = nts.getNetwork();
        // now you have "cn", a CyNetwork instance to do some tests with
```

```
    }
}
```

At some point, while running tests, a `NoClassDefFoundError` might occur, for example:

```
org/cytoscape/event/DummyCyEventHelper
java.lang.NoClassDefFoundError
at ...
at ...
at ...
...
```

This means that some class that was available at compile-time is not longer available while running tests. In this example the missing class is `DummyCyEventHelper`. To resolve this problem, add the package containing the missing class as a test depency:

```
<dependency>
  <groupId>org.cytoscape</groupId>
  <artifactId>event-api</artifactId>
  <version>3.1.0</version>
  <type>test-jar</type>
  <scope>test</scope>
</dependency>
```

A.3.4 Adding a layout algorithm

First of all, two Cytoscape dependencies are required:

```
<dependency>
  <groupId>org.cytoscape</groupId>
  <artifactId>layout-api</artifactId>
  <version>3.1.0</version>
  <scope>provided</scope>
</dependency>
<dependency>
  <groupId>org.cytoscape</groupId>
  <artifactId>work-api</artifactId>
  <version>3.1.0</version>
  <scope>provided</scope>
</dependency>
```

Then, a layout class must be created, which extends `AbstractLayoutAlgorithm`, the class should contain at least the methods shown below:

```
package com.something.package_name;

import java.util.HashSet;
import java.util.Set;

import org.cytoscape.model.CyNode;
import org.cytoscape.view.layout.AbstractLayoutAlgorithm;
import org.cytoscape.view.model.CyNetworkView;
```

```java
import org.cytoscape.view.model.View;
import org.cytoscape.work.TaskIterator;
import org.cytoscape.work.undo.UndoSupport;

public class SomeLayout extends AbstractLayoutAlgorithm {

    /**
     * Creates a new SomeLayout object.
     * @param undo
     */
    public FamLayout(UndoSupport undo) {
        // the two Strings here are the name that can be used
        // to refer to the layout algorithm from source code
        // and the name that will appear in the layouts menu
        // in Cytoscape respectively
        super("computer_layout_name",
              "human_layout_name",
              undo);
    }

    /**
     * Using the method signature as shown here, the
     * layout algorithm can use data of a node attribute
     * to calculate the layout
     */
    public TaskIterator createTaskIterator(CyNetworkView networkView,
        Object context,
        Set<View<CyNode>> nodesToLayOut,
        String attrName) {
        return new TaskIterator(new SomeLayoutTask(toString(),
            networkView,
            nodesToLayOut,
            (SomeLayoutContext) context,
            attrName, // = which node attribute should be used to
                      // perform the grouping on
            undoSupport));
    }

    @Override
    public Set<Class<?>> getSupportedNodeAttributeTypes() {
        Set<Class<?>> ret = new HashSet<Class<?>>();

        ret.add(Integer.class);
        ret.add(Double.class);
        ret.add(String.class);
        ret.add(Boolean.class);
        // add other classes if the layout can support it

        return ret;
    }

    @Override
    public SomeLayoutContext createLayoutContext() {
        return new SomeLayoutContext();
    }

    @Override
```

```
    public boolean getSupportsSelectedOnly() {
        // return false if the layout algorithm can not work on a
        // set of selected nodes, but only on all nodes at once
        return true;
    }

}
```

The class above does not perform the layout, but is used by Cytoscape to get an instance of a `SomeLayoutTask` which does the actual work. The second class that must be created is this task:

```
package com.something.package_name;

import org.cytoscape.model.CyNetwork;
import org.cytoscape.model.CyNode;
import org.cytoscape.model.CyTable;
import org.cytoscape.view.layout.AbstractLayoutTask;
import org.cytoscape.view.model.CyNetworkView;
import org.cytoscape.view.model.View;
import org.cytoscape.view.presentation.property.BasicVisualLexicon;
import org.cytoscape.work.TaskMonitor;
import org.cytoscape.work.undo.UndoSupport;

public class SomeLayoutTask extends AbstractLayoutTask {

    private TaskMonitor taskMonitor;
    private CyNetwork network;
    private SomeLayoutContext context;

    public SomeLayoutTask(final String displayName,
        CyNetworkView networkView,
        Set<View<CyNode>> nodesToLayOut,
        SomeLayoutContext context,
        String attrName,
        UndoSupport undo) {

        super(displayName, networkView, nodesToLayOut, attrName, undo);

        this.context = context;
    }

    /**
     * This method is called by Cytoscape to perform the layout
     */
    @Override
    final protected void doLayout(final TaskMonitor taskMonitor) {
        // here starts the actual work of placing the nodes
        // to get the node instances do:
        for (View<CyNode> nv : nodesToLayOut) {
            CyNode node = nv.getModel();
            // 'node' is now a CyNode instance, to place it at
            // a certain coordinate do:
            networkView.getNodeView(node).setVisualProperty(
                BasicVisualLexicon.NODE_X_LOCATION, x);
            networkView.getNodeView(node).setVisualProperty(
```

```
                BasicVisualLexicon.NODE_Y_LOCATION, y);
            // where 'x' and 'y' are double primitives
        }
    }
}
```

The third and last class is `SomeLayoutContext`, this class contains the options for the layout task. By adding the `@Tunable` annotation, the options will appear in the Cytoscape layout settings window, where the end user can change their values. A minimal example:

```
package com.something.package_name;

import org.cytoscape.work.Tunable;

public class SomeLayoutContext {

    // the values specified here are the default values

    @Tunable(description = "A String option")
    public String someStringOption = "string_option_value";
    @Tunable(description = "A double option, e.g. to set minimal
       distance between nodes")
    public double nodeMinSpacing = 20.0;
    // ad as many options as you like
}
```

Whenever an end user uses a layout algorithm, an instance of this context class with the values as specified by the end user is passed to the constructor of the layout task. This context object can then be used in the layout task code to retrieve the options.

Finally, to add the layout to the Cytoscape layouts menu, put the following code in the `CyActivator` start method. Also, make sure that the package containing the layout code is included in the `Export-Package` tag of the Apache felix setup in the `pom.xml` (see **appendix A.3.1**).

```
UndoSupport undoSupport = getService(context, UndoSupport.class);
SomeLayout layout = new SomeLayout(undoSupport);
Properties layoutProperties = new Properties();
layoutProperties.setProperty("preferredTaskManager", "menu");
// 'TITLE' is inhereted from 'ServiceProperties' by
// 'AbstractCyActivator' and is a String with value: "title"
// The 'toString()' method is defined in AbstractLayoutAlgorithm
// in the Cytoscape layout api and returns the human name of the
// layout algorithm (see above). Thus, the line below sets the
// layout name in the menu to the 'human name'
layoutProperties.setProperty(TITLE, layout.toString());
layoutProperties.setProperty(PREFERRED_MENU, "name of menu entry");
// 'MENU_GRAVITY' is inhereted from 'ServiceProperties' as well.
// The higher the number, the lower the entry appears in the menu
layoutProperties.setProperty(MENU_GRAVITY, "10");
registerService(bundleContext, layout, CyLayoutAlgorithm.class,
    layoutProperties);
```

To apply the layout programatically, get the references to following objects in the start method of the `CyActivator` and pass them to the class that should apply the layout algorithm.

```
TaskManager taskManager = getService(context, TaskManager.class);
CyLayoutAlgorithmManager cyLayoutAlgorithmManager = getService(context,
    CyLayoutAlgorithmManager.class);
```

Use the following code snippet to eventually apply the layout:

```
// get a reference to the network you which to lay-out
CyNetworkView cyNetworkView = ...
// the node attribute used to apply the layout with
String attrName = ...

SomeLayout layout = (SomeLayout) cyLayoutAlgorithmManager().getLayout("
    computer_layout_name");
TaskIterator ti = layout.createTaskIterator(cyNetworkView,
    // below the default options are used, but you can
    // change some values of the context first
    layout.createLayoutContext(),
    CyLayoutAlgorithm.ALL_NODE_VIEWS,
    attrName);
taskManager().execute(ti);
```

A.4 Development workflow

This section contains some suggestions on how to optimize the code-compile-test cycle for writing Cytoscape apps. These suggestion are not the "best" way to do it, but they work well for us. We have used the NetBeans Integrated Development Environment (IDE) (`https://netbeans.org`) which is a popular IDE for java. Another maybe more popular choice is the Eclipse IDE (`https://eclipse.org`). Both IDEs are very similar, so suggestions here are probably equally valid for both NetBeans and Eclipse.

Using NetBeans has many advantages, since NetBeans is aware of the Maven directory structure and dependency mechanism. NetBeans also integrates with Git, and marks changes since the last git commit in the code line numbers, making it really easy to track or revert changes. NetBeans also comes with a graphical debugger and built-in mechanism for executing unit tests among many other features.

Importing a Maven project into Netbeans To import a Maven project, click *File→Open Project* and navigate to the folder containing the maven project. Select the folder and click *Open Project*. NetBeans will automatically detect that the project is a maven project, and if a `.git` directory is present, NetBeans will automatically integrate with Git.

Code-compile-test Start Cytoscape from the command line, also open a `tail` for the Cytoscape log file in another terminal window. This enables control over which OSGi bundles are activated, and gives information about eventual exceptions/errors and OSGi

output. Print statements to `STDOUT` from bundles are printed to the console, whenever a new bundle is activated, it is printed to the log file.

```
$ ./Cytoscape_vx.x.0/cytoscape.sh
$ # in another terminal window
$ tail -f CytoscapeConfiguration/3/framework-cytoscape.log
```

Also, as mentioned in **appendix A.1.3**, symlinking the `.jar` file to the Cytoscape apps folder will make Cytoscape to automatically update the plugin whenever it is recompiled. Thus, it is almost never needed to restart Cytoscape while working on a plugin.

```
$ ln -s $PLUGIN_ROOT/CoExpNetViz/target/CoExpNetViz-1.0-SNAPSHOT.jar
    $CYTOSCAPE_HOME/CytoscapeConfiguration/3/apps/installed
```

When clicking the *Clean And Build* button, or pressing `shift-F11` the Maven goal `clean install` is executed, and Maven output from executing goals is printed to an output window in the IDE. Thus, in a typical workflow, you modified some code, then hit `shift-F11` and see if the result is as expected in Cytoscape, this way, you can check the effect of the code changes in a matter of seconds.

To test the currently open source file, hit `shift-F6`, or to execute all JUnit tests, hit `alt-F6`. If there are test files present in the default testing directory (see **appendix A.3.3**), the `maven-surefire-plugin` will perform the JUnit tests. When using version 2.15 or higher of the surefire plugin, individual methods can be tested as well. This provides a quick way to test out code without having to use Cytoscape.

A.4.1 Debugging the plugin code

Cytoscape can started from the command line with the option "`debug`", this will print the following output to the terminal:

```
$ ./Cytoscape_vx.x.0/cytoscape.sh debug
Listening for transport dt_socket at address: 12345
```

Then, in NetBeans, click *Degug→Attach Debugger*, this will open a dialog asking for a socket address. Choose the options as shown in **fig. A.1**, except for the hostname, which is the name of the computer.

After clicking *OK*, Cytoscape will start, and every thread started by Cytoscape can be seen in the IDE window. In addition, it is possible to place breakpoints in the plugin source code, and step trough the code line-by-line, every variable value can checked, and watches can be created to to check how one or more variables changes while the program is running.

If only a very limited amount of source code was changed, clicking the "*Apply Code Changes*" button will apply the effect of the change immediately without even recompiling the plugin. This can be very useful for processes that require many trial-and-error runs, as, for example, placing GUI components in aesthetically pleasing positions. But whenever a method definition is changed/added/deleted, *Apply Code Changes* will not longer work, and recompiling the app will be necessary. After recompiling, the Netbeans debugger can point to incorrect line numbers while stepping trough source code. This can be solved quickly be stopping the debugging session, Cytoscape will just keep running while waiting

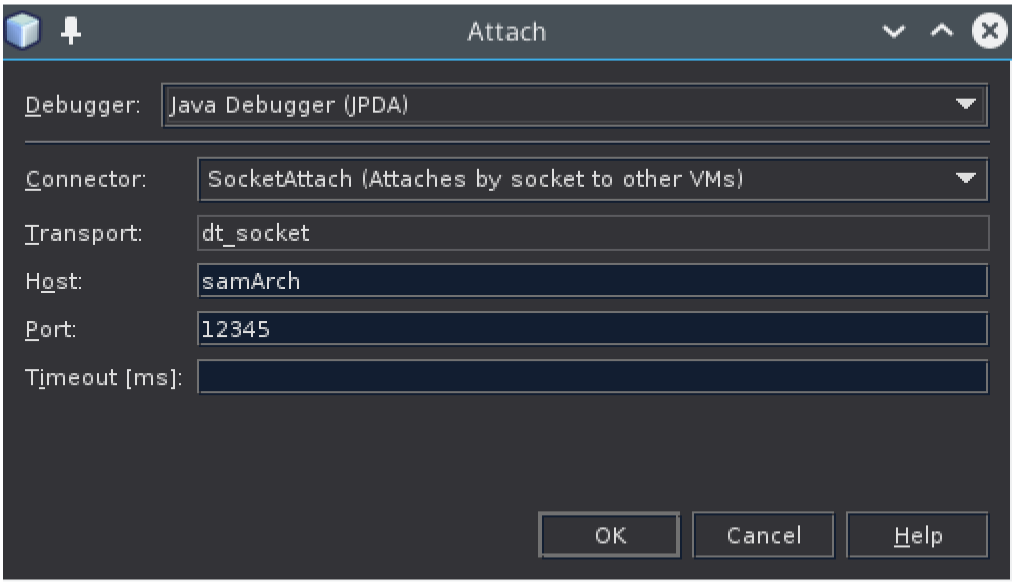

Figure A.1: Attaching the NetBeans debugger to CytoScape. This window can look different depending on which operating system is used.

for a new debugger to attach. Then, re-attach the debugger in Netbeans and continue working as before.

A.4.2 Stepping into the Cytoscape core program source code

The Cytoscape API is not perfect, and sometimes, bugs in the plugin are happening due to bugs in the Cytoscape API. In that case, it can help to take a look into the Cytoscape source code to see where the problem arose. There is no better way to explore the source code of a large program such as Cytoscape then by compiling it yourself, and running it with a graphical debugger attached.

Adequate instructions to compile Cytoscape from source are available on the Cytoscape GitHub page (`https://github.com/cytoscape/cytoscape`). Many dependencies will be downloaded when compiling Cytoscape for the first time, which can result in a compile time of up to two hours. After the first compilation, when all dependencies are already met, the compilation will take between five and fifteen minutes depending on the hardware of the computer.

The Cytoscape Maven parent project can be imported into NetBeans in the same way as explained in **appendix A.4**. Once the Cytoscape project is imported into NetBeans and compiled either from the command line or from within NetBeans, it can be started in debug mode in the same way as explained in **appendix A.4.1**. Everything the graphical debugger has to offer, such as placing breakpoints, watching variables, etc. is now also possible for the Cytoscape source code, this can be very helpful to solve bugs that originated from within the Cytoscape API.

Appendix B

CoExpNetViz user manual

Foreword

This is the CoExpNetViz user manual, which is also available at the CoExpNetViz website. See also the Github repository `https://github.com/SamDM/CoExpNetViz` and **appendix A** for development information.

B.1 Installation

The normal way to install a Cytoscape plugin is with the *App Manager*, but, as CoExpNetViz is not yet published, this installation method is unavailable. To install the plugin, download the program from the CoExpNetViz website at `http://bioinformatics.psb.ugent.be/webtools/coexpr/index.php` and copy it to the folder:

```
$CYTOSCAPE_HOME/CytoscapeConfiguration/3/apps/installed
```

In most cases, the `$CYTOSCAPE_HOME` directory, which is the directory where Cytoscape is installed, is located in the user home folder. If Cytoscape is already running, the app can be used immediately, if not, then the next time Cytoscape is started, the app will be installed.

Important To run the app, Cytoscape version 3.1 or higher should be used. It is strongly recommended to use java 1.6 or 1.7 to run Cytoscape. Java 1.8 might also appear to work fine, but can cause strange behavior in Cytoscape.

B.2 Usage of CoExpNetViz

To start the app, go to *Apps→CoExpNetViz* in the Cytoscape menu, clicking this entry will launch a form where gene expression datasets can be submitted.

To find out which genes are coexpressed with your genes of interest, enter these genes in the *bait genes* text field (2 in **fig. B.1**) or, alternatively, upload a file with bait genes (3 in **fig. B.1**, see also **appendix B.5** for file formats).

The next step is choosing gene expression datasets, to enter a dataset, click the *browse* button (4b in **fig. B.1**) and navigate to the file, for the *Species* field (4a in **fig. B.1**), any name can be chosen. To add additional datasets, click the *Add species* button (5 in **fig. B.1**), a maximum of five species can be used at once.

Then, choose PCC cutoff values (6 in **fig. B.1**), to include only positive or negative correlations, set the negative cutoff to -1.0 or positive the cutoff 1.0 respectively.

If you which to save the output of the analysis, check the box *Save output* (7 in **fig. B.1**), the output will be saved as a `*.tar.gz` archive in the specified folder. The name of the file will be the *Title* (1 in **fig. B.1**).

Finally, to run the analysis, click the *Run analysis* button (8 in **fig. B.1**), CoExpNetViz will then run the analysis on the web server. Depending on your internet speed and the size of the gene expression files, this can take about ten seconds up to a few minutes. When the analysis is complete, a new network will appear in Cytoscape.

Specifying custom gene families In addition to the options described above, custom gene families can be used to define orthologous genes. By default, CoExpNetViz will use PLAZA families to find orthologous genes, but by specifying your own orthologous genes, other gene families, such as Ortho-MCL families, can be used as well. To specify other gene families, go to the *Gene family options* tab (9 in **fig. B.1**), there, you can choose up to five gene family files to be used (the interface for this is very similar to 4 and 5 in **fig. B.1**).

CoExpNetViz will merge gene families (from the same file or across submitted gene family files) if they contain the same gene. Also, CoExpNetViz already has the PLAZA monocot and PLAZA dicot families in memory by default. So merging will occur with these families as well if the submitted gene family files contain ID's that are also present in PLAZA.

B.3 The family wise coexpression graph

If the analysis run succesfully, a new graph can be seen in the Cytoscape main window: the family wise coexpression graph (**fig. B.2**). In the default layout, bait genes are placed at the corners of the graph as big white diamond shaped nodes. The nodes in the middle are gene families, and the links between gene families and baits are coexpression relationships (the PCC of at least one gene in this family to the bait is greater than the given threshold). Positive correlations are shown in blue, while negative correlations are shown in red. Additionally, homology relationships between bait genes are shown as light-yellow dotted lines. In the default layout, target genes are grouped into partitions, where every partition has its own color. Nodes in the same partition have links to same set of baits.

To get more information about a node, right click the node and choose *Apps→CoExpNetViz* (**fig. B.5a**). A small dialog window will present links that bring you to the gene family web pages, (**fig. B.5b**) to close the window, click the `x` at the top right or hit `esc`.

B.4 Web interface

CoExpNetViz is also available as a web tool on `http://bioinformatics.psb.ugent.be/webtools/coexpr/index.php`. After running the analysis on the web interface, the output files can be downloaded and imported into Cytoscape manually. The input form on the web interface is very similar to the plugin but currently (as of June 8, 2015), the web interface is still a work in progress, it is therefore not discussed here. This section might be completed in the future.

B.5 File formats

Example bait genes files, gene expression files and gene family files can downloaded from the CoExpNetViz website.

Bait genes For the bait genes file, a plain text file is expected with gene ID's separated by any kind of whitespace. For information about supported gene identifiers, click the *ID's* button (11 in **fig. B.1**).

Gene expression files The gene expression files should have a matrix format, with all gene ID's in the first column and all conditions in the first row. Columns must be tab separated. The expression data must be normalized and summarized, this means the data must be background corrected, normalized across conditions and summarized to gene expression values (not probe intensities). Also, if you which to use log transformed

data, you must first do the log transform yourself, as CoExpNetViz will not transform or alter the data in any way. Finally, avoid having many similar conditions in the microarray dataset, this will lead to columns with redundant information and many very high PCCs

Gene family files The gene family files must follow the following format: every line starts with the name of a gene family, followed by a tab character and a list of genes belonging to that family, the genes are also tab separated. The file may not contain a column header.

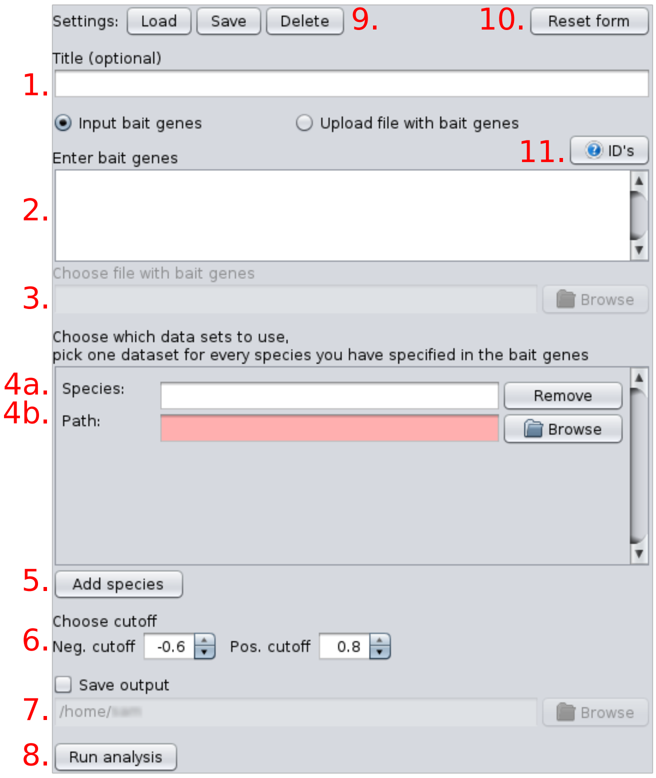

Figure B.1: CoExpNetViz input form. Fill in the form and click *Run analysis* (8) to start the coexpression analysis. To save a specific configuration, click *Save*, then click *Load* to load it again later or click *Delete* to remove the saved configuration (10). To clear all input, click *Reset form* (11). For more information about which gene ID's can be used, click *ID's* (12).

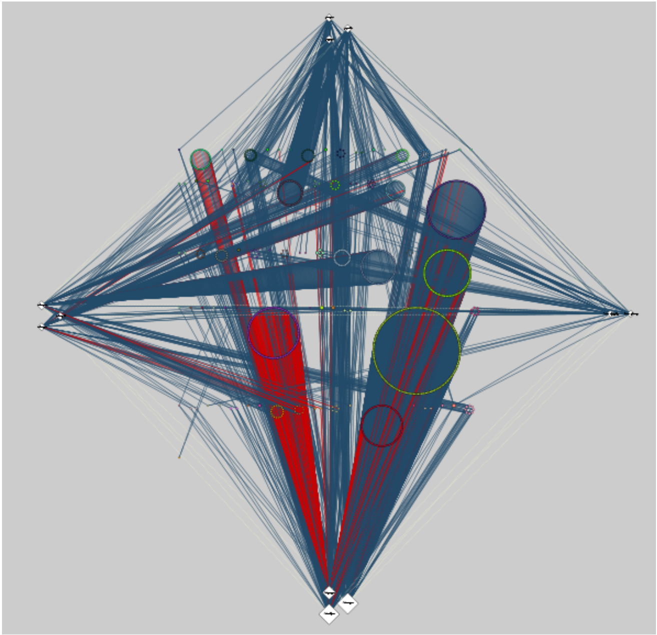

Figure B.2: Overview of a family wise coexpression graph. This graph was created by using 11 bait genes from four different species, along with four gene expression datasets, one for each species. The positive cutoff was set to 0.8 and the negative cutoff was set to -0.6.

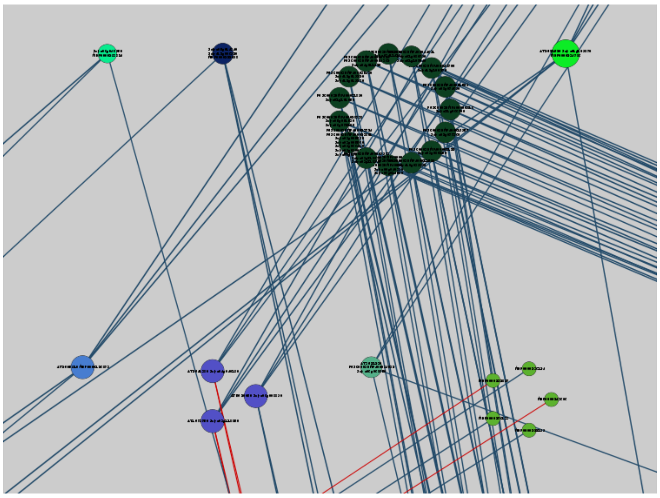

Figure B.3: Detail of the gene families. The upper right node (bright green) is a family containing genes that are coexpressed with 7 out of the 11 bait genes, and is likely involved in the same biological process as the bait genes. Gene names of the coexpressed genes are displayed on top of each node.

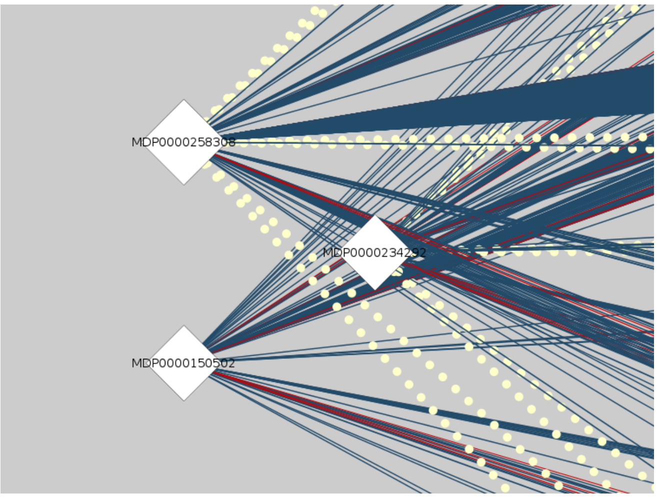

Figure B.4: Detail of the bait genes. A close up of the *Malus domesticus* (apple) bait genes is shown.

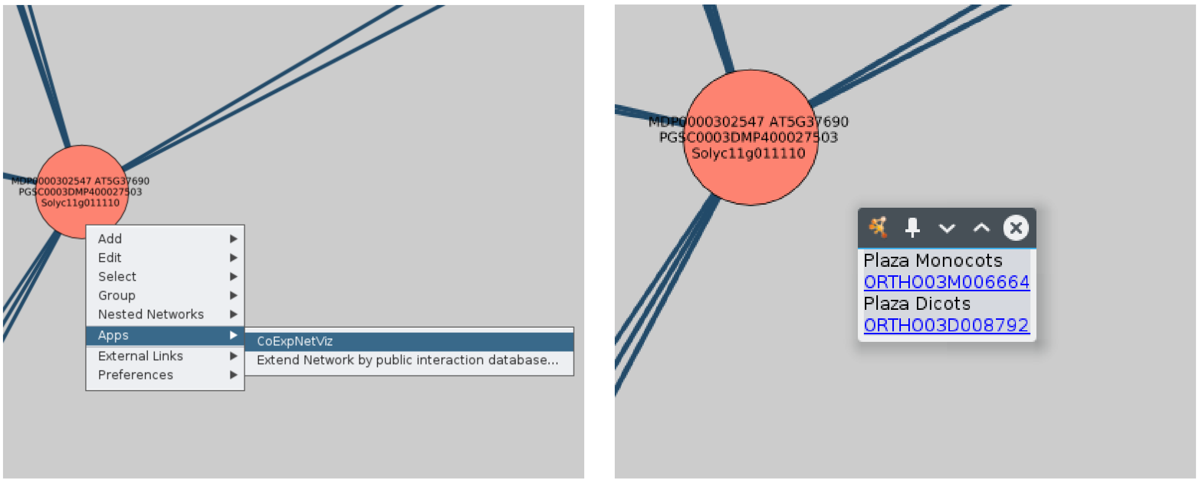

(a) Using the context menu (b) Linkouts to PLAZA families

Figure B.5: Linking gene families to the PLAZA website.

B.6 How to submit bugs

If you encountered a bug, you can submit it at the CoExpNetViz Github page at `https://github.com/CoExpNetViz` or you can send an email to contact.person@placeholder.org.

Bibliography

Altschul, S.F., Gish, W., Miller, W., Myers, E.W. and Lipman, D.J. Basic local alignment search tool. *Journal of molecular biology*, 215(3):403–10, October 1990. ISSN 0022-2836. doi: 10.1016/S0022-2836(05)80360-2. URL http://www.ncbi.nlm.nih.gov/pubmed/2231712.

Ashburner, M., Ball, C.A., Blake, J.A., Botstein, D., Butler, H. *et al.* Gene ontology: tool for the unification of biology. The Gene Ontology Consortium. *Nature genetics*, 25 (1):25–9, May 2000. ISSN 1061-4036. doi: 10.1038/75556. URL http://dx.doi.org/10.1038/75556.

Barrett, T., Wilhite, S.E., Ledoux, P., Evangelista, C., Kim, I.F. *et al.* NCBI GEO: archive for functional genomics data sets–update. *Nucleic acids research*, 41(Database issue):D991–5, January 2013. ISSN 1362-4962. doi: 10.1093/nar/gks1193. URL http://nar.oxfordjournals.org/content/41/D1/D991.full.

Bergmann, S., Ihmels, J. and Barkai, N. Similarities and differences in genome-wide expression data of six organisms. *PLoS biology*, 2(1):E9, January 2004. ISSN 1545-7885. doi: 10.1371/journal.pbio.0020009. URL http://journals.plos.org/plosbiology/article?id=10.1371/journal.pbio.0020009.

Croft, D., Mundo, A.F., Haw, R., Milacic, M., Weiser, J. *et al.* The Reactome pathway knowledgebase. *Nucleic acids research*, 42(Database issue):D472–7, January 2014. ISSN 1362-4962. doi: 10.1093/nar/gkt1102. URL http://www.pubmedcentral.nih.gov/articlerender.fcgi?artid=3965010&tool=pmcentrez&rendertype=abstract.

Daub, C.O., Steuer, R., Selbig, J. and Kloska, S. Estimating mutual information using B-spline functions–an improved similarity measure for analysing gene expression data. *BMC bioinformatics*, 5:118, August 2004. ISSN 1471-2105. doi: 10.1186/1471-2105-5-118. URL http://www.pubmedcentral.nih.gov/articlerender.fcgi?artid=516800&tool=pmcentrez&rendertype=abstract.

De Bodt, S., Hollunder, J., Nelissen, H., Meulemeester, N. and Inzé, D. CORNET 2.0: integrating plant coexpression, protein-protein interactions, regulatory interactions, gene associations and functional annotations. *The New phytologist*, 195(3): 707–20, August 2012. ISSN 1469-8137. doi: 10.1111/j.1469-8137.2012.04184.x. URL http://www.ncbi.nlm.nih.gov/pubmed/22651224.

Enright, A.J., Van Dongen, S. and Ouzounis, C.A. An efficient algorithm for large-scale detection of protein families. *Nucleic acids research*, 30(7):1575–84, April 2002. ISSN 1362-4962. URL http://www.pubmedcentral.nih.gov/articlerender.fcgi?artid=101833&tool=pmcentrez&rendertype=abstract.

Hansen, B.O., Vaid, N., Musialak-Lange, M., Janowski, M. and Mutwil, M. Elucidating gene function and function evolution through comparison of co-expression networks of plants. *Frontiers in Plant Science*, 5(August):1–9, 2014. ISSN 1664-462X. doi: 10.3389/fpls.2014.00394. URL http://www.frontiersin.org/Plant_Science/10.3389/fpls.2014.00394/abstract.

Kanehisa, M., Goto, S., Sato, Y., Kawashima, M., Furumichi, M. *et al.* Data, information, knowledge and principle: back to metabolism in KEGG. *Nucleic acids research*, 42(Database issue):D199–205, January 2014. ISSN 1362-4962. doi: 10.1093/nar/gkt1076. URL http://www.pubmedcentral.nih.gov/articlerender.fcgi?artid=3965122&tool=pmcentrez&rendertype=abstract.

Li, L., Stoeckert, C.J. and Roos, D.S. OrthoMCL: identification of ortholog groups for eukaryotic genomes. *Genome research*, 13(9):2178–89, September 2003. ISSN 1088-9051. doi: 10.1101/gr.1224503. URL http://www.pubmedcentral.nih.gov/articlerender.fcgi?artid=403725&tool=pmcentrez&rendertype=abstract.

Maere, S., Heymans, K. and Kuiper, M. BiNGO: a Cytoscape plugin to assess overrepresentation of gene ontology categories in biological networks. *Bioinformatics (Oxford, England)*, 21(16):3448–9, August 2005. ISSN 1367-4803. doi: 10.1093/bioinformatics/bti551. URL http://www.ncbi.nlm.nih.gov/pubmed/15972284.

Mewalal, R., Mizrachi, E., Mansfield, S.D. and Myburg, A.A. Cell Wall-Related Proteins of Unknown Function: Missing Links in Plant Cell Wall Development. *Plant and Cell Physiology*, 55(6):1031–1043, March 2014. ISSN 0032-0781. doi: 10.1093/pcp/pcu050. URL http://www.ncbi.nlm.nih.gov/pubmed/24683037.

Movahedi, S., Van Bel, M., Heyndrickx, K.S. and Vandepoele, K. Comparative co-expression analysis in plant biology. *Plant, cell & environment*, 35(10):1787–98, October 2012. ISSN 1365-3040. doi: 10.1111/j.1365-3040.2012.02517.x. URL http://www.ncbi.nlm.nih.gov/pubmed/22489681.

Mutwil, M., Obro, J., Willats, W.G.T. and Persson, S. GeneCAT–novel webtools that combine BLAST and co-expression analyses. *Nucleic acids research*, 36 (Web Server issue):W320–6, July 2008. ISSN 1362-4962. doi: 10.1093/nar/gkn292. URL http://www.pubmedcentral.nih.gov/articlerender.fcgi?artid=2447783&tool=pmcentrez&rendertype=abstract.

Mutwil, M., Klie, S., Tohge, T., Giorgi, F.M., Wilkins, O. *et al.* PlaNet: combined sequence and expression comparisons across plant networks derived from seven species. *The Plant cell*, 23(3):895–910, March 2011. ISSN 1532-298X. doi: 10.1105/tpc.111.083667. URL http://www.pubmedcentral.nih.gov/articlerender.fcgi?artid=3082271&tool=pmcentrez&rendertype=abstract.

Proost, S., Van Bel, M., Vaneechoutte, D., Van de Peer, Y., Inzé, D. *et al.* PLAZA 3.0: an access point for plant comparative genomics. *Nucleic acids research*, 43(Database issue):D974–81, January 2015. ISSN 1362-4962. doi: 10.1093/nar/gku986. URL http://nar.oxfordjournals.org/content/43/D1/D974.

Rasmussen, S., Barah, P., Suarez-Rodriguez, M.C., Bressendorff, S., Friis, P. *et al.* Transcriptome responses to combinations of stresses in Arabidopsis. *Plant physiology*, 161(4):1783–94, April 2013. ISSN 1532-2548. doi: 10.1104/pp.112.210773. URL http://www.pubmedcentral.nih.gov/articlerender.fcgi?artid=3613455&tool=pmcentrez&rendertype=abstract.

Rhee, S.Y. and Mutwil, M. Towards revealing the functions of all genes in plants. *Trends in plant science*, 19(4):212–21, April 2014. ISSN 1878-4372. doi: 10.1016/j.tplants.2013.10.006. URL http://www.sciencedirect.com/science/article/pii/S1360138513002343.

Saito, R., Smoot, M.E., Ono, K., Ruscheinski, J., Wang, P.L. *et al.* A travel guide to Cytoscape plugins. *Nature methods*, 9(11):1069–76, November 2012. ISSN 1548-7105. doi: 10.1038/nmeth.2212. URL http://www.pubmedcentral.nih.gov/articlerender.fcgi?artid=3649846&tool=pmcentrez&rendertype=abstract.

Sewelam, N., Oshima, Y., Mitsuda, N. and Ohme-Takagi, M. A step towards understanding plant responses to multiple environmental stresses: a genome-wide study. *Plant, cell & environment*, 37(9):2024–35, September 2014. ISSN 1365-3040. doi: 10.1111/pce.12274. URL http://www.ncbi.nlm.nih.gov/pubmed/24417440.

Shannon, P., Markiel, A., Ozier, O., Baliga, N.S., Wang, J.T. *et al.* Cytoscape: a software environment for integrated models of biomolecular interaction networks. *Genome research*, 13(11):2498–504, November 2003. ISSN 1088-9051. doi: 10.1101/gr.1239303. URL http://www.pubmedcentral.nih.gov/articlerender.fcgi?artid=403769&tool=pmcentrez&rendertype=abstract.

Steinhauser, D., Usadel, B., Luedemann, A., Thimm, O. and Kopka, J. CSB.DB: a comprehensive systems-biology database. *Bioinformatics*, 20(18):3647–3651, July 2004. ISSN 1367-4803. doi: 10.1093/bioinformatics/bth398. URL http://www.ncbi.nlm.nih.gov/pubmed/15247097.

Steuer, R., Kurths, J., Daub, C.O., Weise, J. and Selbig, J. The mutual information: detecting and evaluating dependencies between variables. *Bioinformatics (Oxford, England)*, 18 Suppl 2:S231–40, January 2002. ISSN 1367-4803. URL http://www.ncbi.nlm.nih.gov/pubmed/12386007.

Stuart, J.M., Segal, E., Koller, D. and Kim, S.K. A gene-coexpression network for global discovery of conserved genetic modules. *Science (New York, N.Y.)*, 302(5643):249–55, October 2003. ISSN 1095-9203. doi: 10.1126/science.1087447. URL http://www.ncbi.nlm.nih.gov/pubmed/12934013.

The Arabidopsis Genome Initiative. Analysis of the genome sequence of the flowering plant Arabidopsis thaliana. *Nature*, 408(6814):796–815, December 2000. ISSN 0028-0836. doi: 10.1038/35048692. URL http://dx.doi.org/10.1038/35048692.

The Potato Genome Sequencing Consortium. Genome sequence and analysis of the tuber crop potato. *Nature*, 475(7355):189–95, July 2011. ISSN 1476-4687. doi: 10.1038/nature10158. URL http://dx.doi.org/10.1038/nature10158.

The Tomato Genome Consortium. The tomato genome sequence provides insights into fleshy fruit evolution. *Nature*, 485(7400):635–41, May 2012. ISSN 1476-4687. doi: 10.1038/nature11119. URL http://dx.doi.org/10.1038/nature11119.

Toufighi, K., Brady, S.M., Austin, R., Ly, E. and Provart, N.J. The Botany Array Resource: e-Northerns, Expression Angling, and promoter analyses. *The Plant journal : for cell and molecular biology*, 43(1):153–63, July 2005. ISSN 0960-7412. doi: 10.1111/j.1365-313X.2005.02437.x. URL http://www.ncbi.nlm.nih.gov/pubmed/15960624.

Usadel, B., Obayashi, T., Mutwil, M., Giorgi, F.M., Bassel, G.W. *et al.* Co-expression tools for plant biology: opportunities for hypothesis generation and caveats. *Plant, cell & environment*, 32(12):1633–51, December 2009a. ISSN 1365-3040. doi: 10.1111/j.1365-3040.2009.02040.x. URL http://www.ncbi.nlm.nih.gov/pubmed/19712066.

Usadel, B., Poree, F., Nagel, A., Lohse, M., Czedik-Eysenberg, A. *et al.* A guide to using MapMan to visualize and compare Omics data in plants: a case study in the crop species, Maize. *Plant, cell & environment*, 32(9):1211–29, September 2009b. ISSN 1365-3040. doi: 10.1111/j.1365-3040.2009.01978.x. URL http://www.ncbi.nlm.nih.gov/pubmed/19389052.

van Dongen, S. Graph Clustering by Flow Simulation. May 2000. URL http://www.narcis.nl/publication/RecordID/oai%3Acwi.nl%3A18026.

Velasco, R., Zharkikh, A., Affourtit, J., Dhingra, A., Cestaro, A. *et al.* The genome of the domesticated apple (Malus × domestica Borkh.). *Nature genetics*, 42(10):833–9, October 2010. ISSN 1546-1718. doi: 10.1038/ng.654. URL http://dx.doi.org/10.1038/ng.654.

Zhang, W., Liu, T., Ren, G., Hörtensteiner, S., Zhou, Y. *et al.* Chlorophyll degradation: the tocopherol biosynthesis-related phytol hydrolase in Arabidopsis seeds is still missing. *Plant physiology*, 166(1):70–9, September 2014. ISSN 1532-2548. doi: 10.1104/pp.114.243709. URL http://www.pubmedcentral.nih.gov/articlerender.fcgi?artid=4149732&tool=pmcentrez&rendertype=abstract.
